# Molecular and clinical characteristics of *ATP1A3*-related diseases

**DOI:** 10.3389/fneur.2022.924788

**Published:** 2022-07-26

**Authors:** Yinchao Li, Xianyue Liu, Chengzhe Wang, Zhengwei Su, Ke Zhao, Man Yang, Shuda Chen, Liemin Zhou

**Affiliations:** ^1^Department of Neurology, The Seven Affiliated Hospital, Sun Yat-sen University, Shenzhen, China; ^2^Department of Neurology, The First Affiliated Hospital, Sun Yat-sen University, Guangzhou, China

**Keywords:** *ATP1A3*, phenotype spectrum, alternating hemiplegia of childhood, flunarizine, rapid-onset dystonia-parkinsonism

## Abstract

**Objective:**

With detailed studies of *ATP1A3*-related diseases, the phenotypic spectrum of *ATP1A3* has greatly expanded. This study aimed to potentially identify the mechanisms by which *ATP1A3* caused neurological dysfunction by analyzing the clinical features and phenotypes of *ATP1A3*-related diseases, and exploring the distribution patterns of mutations in the subregions of the *ATP1A3* protein, thus providing new and effective therapeutic approaches.

**Methods:**

Databases of PubMed, Online Mendelian Inheritance in Man, and Human Gene Mutation Database, Wanfang Data, and Embase were searched for case reports of *ATP1A3*-related diseases. Following case screening, we collected clinical information and genetic testing results of patients, and analyzed the disease characteristics on the clinical phenotype spectrum associated with mutations, genetic characteristics of mutations, and effects of drug therapy.

**Results:**

We collected 902 clinical cases related to *ATP1A3* gene. From the results of previous studies, we further clarified the clinical characteristics of *ATP1A3-*related diseases, such as alternating hemiplegia of childhood (AHC), rapid-onset dystonia-parkinsonism; cerebellar ataxia, areflexia, pes cavus, optic atrophy, and sensorineural hearing loss syndrome, and relapsing encephalopathy with cerebellar ataxia, frequency of mutations in different phenotypes and their distribution in gene and protein structures, and differences in mutations in different clinical phenotypes. Regarding the efficacy of drug treatment, 80 of the 124 patients with AHC were treated with flunarizine, with an effectiveness rate of ~64.5%.

**Conclusions:**

Nervous system dysfunction due to mutations of *ATP1A3 gene* was characterized by a group of genotypic–phenotypic interrelated disease pedigrees with multiple clinical manifestations. The presented results might help guide the diagnosis and treatment of *ATP1A3*-related diseases and provided new ideas for further exploring the mechanisms of nervous system diseases due to *ATP1A3* mutations.

## Introduction

*ATP1A3* contains 23 exons and 22 introns, and encodes a protein of 1013 amino acids, consisting of the α3 subunit of Na^+^/K^+^-ATPase, which is located on chromosome 19q13.2 and is expressed primarily in brain neuronal cells (1). Na^+^/K^+^-ATPase is a membrane-binding transporter with ATP hydrolase activity, which is mainly expressed in neuronal cells. It is a key transmembrane protein that transports sodium and potassium ions in the neuronal membrane and ultimately regulates brain function (2). Comprehensive studies have revealed that Na^+^/K^+^-ATPase was not only related to ion transport but also helped regulate proton transport (3, 4). Na^+^/K^+^-ATPase utilized energy from ATP hydrolysis to transport three Na^+^ to the outside of the cell in exchange for two extracellular K^+^ and maintained the acid–base balance inside and outside the cell and, in turn, regulated neuronal excitability with the passive inward transport of protons (4). An increasing number of studies has demonstrated that some *ATP1A3* mutations could lead to a range of clinical phenotypes, including alternating hemiplegia of childhood (AHC); rapid-onset dystonia-parkinsonism (RDP); cerebellar ataxia, areflexia, pes cavus, optic atrophy, and sensorineural hearing loss (CAPOS) syndrome; epilepsy; relapsing encephalopathy with cerebellar ataxia (RECA); fever-induced paroxysmal weakness and encephalopathy (FIPWE); early infantile epileptic encephalopathy (EIEE) and childhood-onset schizophrenia (COS). The currently identified clinical phenotypes of neurological disorders associated with *ATP1A3* mutations were overlapping but not completely consistent.

Case reports of mixed or intermediate phenotypes among disorders have demonstrated that the nervous system dysfunction caused by *ATP1A3* mutation manifests as a group of genotype–phenotype interrelated disease spectra with various clinical manifestations. The diverse phenotypes involved in neurological attacks included dystonia, hemiparesis, ataxia, epilepsy, abnormal eye movement, and bulbar symptoms, such as dysphagia and dysarthria, as well as permanent cognitive impairment, motor dysfunction, and emotional and behavioral disorders. Therefore, further analysis of the clinical characteristics of *ATP1A3* and the genotype–phenotype relationship helped in the clinical diagnosis and treatment of neurological diseases. With further investigation of *ATP1A3*-related diseases, the phenotypic spectra of *ATP1A3* may be further expanded.

Therefore, this study analyzed the clinical characteristics of *ATP1A3*-related diseases and the relationship between genotype and phenotype by considering the results of previous studies on *ATP1A3* mutation-related diseases and two cases in our center. Analyzing the common characteristics of diseases in the phenotypic spectra may provide new ideas for exploring the mechanism of *ATP1A3-*induced neurological dysfunctions. The results will provide new clues and effective therapeutic targets for disease prevention and treatment.

## Materials and methods

### Study patients

#### Source of enrolled patients

Databases of PubMed, Online Mendelian Inheritance in Man, Human Gene Mutation Database, Chinese National Knowledge Infrastructure, Wanfang Data, and Embase were searched for case reports related to *ATP1A3* from the time of database establishment to August 2020. The search subject term was “*ATP1A3* protein, human,” and the search keywords were as follows: “Sodium-Potassium-Exchanging ATPase,” “DYT12 protein, human,” “sodium-potassium ATPase catalytic subunit alpha-3 protein, human,” “ATPase, Na+-K+ transporting, alpha 3 polypeptide, human,” “dystonia 12 protein, human,” and “sodium pump subunit alpha-3 protein, human.”

### Inclusion criteria and exclusion criteria

#### Inclusion criteria

All the *ATP1A3* mutation-positive participants were included in this study.

#### Exclusion criteria

(1) Gene sequencing found mutations in other related genes that caused the disease and could explain the patient's current clinical manifestations.

(2) Reports where clinical information was incomplete or resulted in significant bias were not included in the genotype–disease characteristics correlation analysis.

(3) The study was not published in English or Chinese.

### Methods

#### Collection of patient data

A clinical registry was established for each patient, which included their ID; sex; onset age; symptoms at onset; other symptoms (hemiplegia, abnormal eye movements, abnormal posture, seizures, paroxysmal kinesigenic choreoathetosis, ataxia, autonomic, etc.); development of language, motor skills, and intelligence; perinatal status; personal history; family history; medication history; and ancillary examination results. For clinical data not mentioned in previous studies, attempts were made to contact the corresponding author to obtain this information.

#### Determining the patient's clinical phenotype

The diagnosis was determined based on the diagnostic and classification criteria for clinical phenotypes (such as AHC, RDP, CAPOS syndrome, and RECA), and the diagnosis and typing of patients in the included studies.

#### Analysis of the distribution characteristics of mutations in the submolecular structure of *ATP1A3*

In this study, the distribution of the mutation sites on the submolecular structure of *ATP1A3* was analyzed to investigate whether there was a specific pattern in the distribution of mutations of different phenotypes in each structural subregion of *ATP1A3*.

#### Statistical methods

The Statistical Package for the Social Sciences version 25.0 software was used for statistical analysis. The enumeration data were expressed in percentage, and the measurement data were expressed as mean ± standard deviation. All statistical tests were two-tailed. The *P*-value for *ATP1A3* gene mutation types and phenotypes difference determined by using Fisher exact test. The *p*-values with *p* < 0.05 were taken as statistically significant.

## Results

### Clinical data

The study enrolled a total of 902 cases, including 638 cases of AHC, 119 of RDP, 54 of CAPOS syndrome, 12 of RECA, 12 of FIPWE, 5 of EIEE, 4 of COS, and 58 of other clinical phenotypes (exhibiting overlapping or other phenotypes). In addition, 352 cases only provided gene mutation and diagnosis information but lacked other clinical information. According to the available information, there were 134 female and 250 male participants. Sex information was not provided in other cases. The patients' age of onset was 28.69 ± 72.41 (0–708) months, and the age at the last evaluation was 17.13 ± 15.03 (0.25–87) years.

#### Clinical phenotypic profile associated with *ATP1A3* mutations

*ATP1A3*-related diseases characteristics were determined by combining information from previous studies and the results of the present study. Given the phenotypic variations of *ATP1A3*-related diseases, the following two points should be noted: (1) The symptoms did not occur in all patients, but they were very common; thus, they were considered important features of the clinical phenotypic spectrum. (2) Although some clinical manifestations were not marked as disease features, they may occured in a few patients with this gene mutation, and they were not included in the disease characteristics because they are not very common.

The main clinical manifestations of patients with the AHC phenotype were abnormal eye movements, bilateral alternating hemiplegia, dystonia, seizures, as well as autonomic nervous symptoms, dyspnea, psychomotor retardation, choreoathetosis, and dystonia. The main manifestations of RDP were bulbar symptoms, such as dysarthria, dysphagia, and dysphagia, often accompanied by dystonia, dyskinesia, and postural instability. CAPOS syndrome was mostly induced by febrile diseases, and its main clinical manifestations were cerebellar ataxia, areflexia, optic atrophy, sensorineural hearing loss, and pes cavus. The few reported cases of RECA, mainly manifesting as ataxia, disturbance of consciousness, dystonia, dysphagia, and speech disorders, were often accompanied by cerebellar atrophy.

FIPWE is an acute attack event often triggered by fever. The disease is mainly characterized by limb weakness and encephalopathy and is often accompanied by involuntary movement, dystonia, dystonia, and tendon reflex weakening or disappearance. Four new clinical phenotypes were reported in recent studies: dystonia, dysmorphism of the face, encephalopathy with developmental delay, and brain magnetic resonance imaging (MRI) abnormalities always including cerebellar hypoplasia, absence of hemiplegia (Ø), and the neonatal onset of symptoms (D-DEMØ).

**Figure 1:**
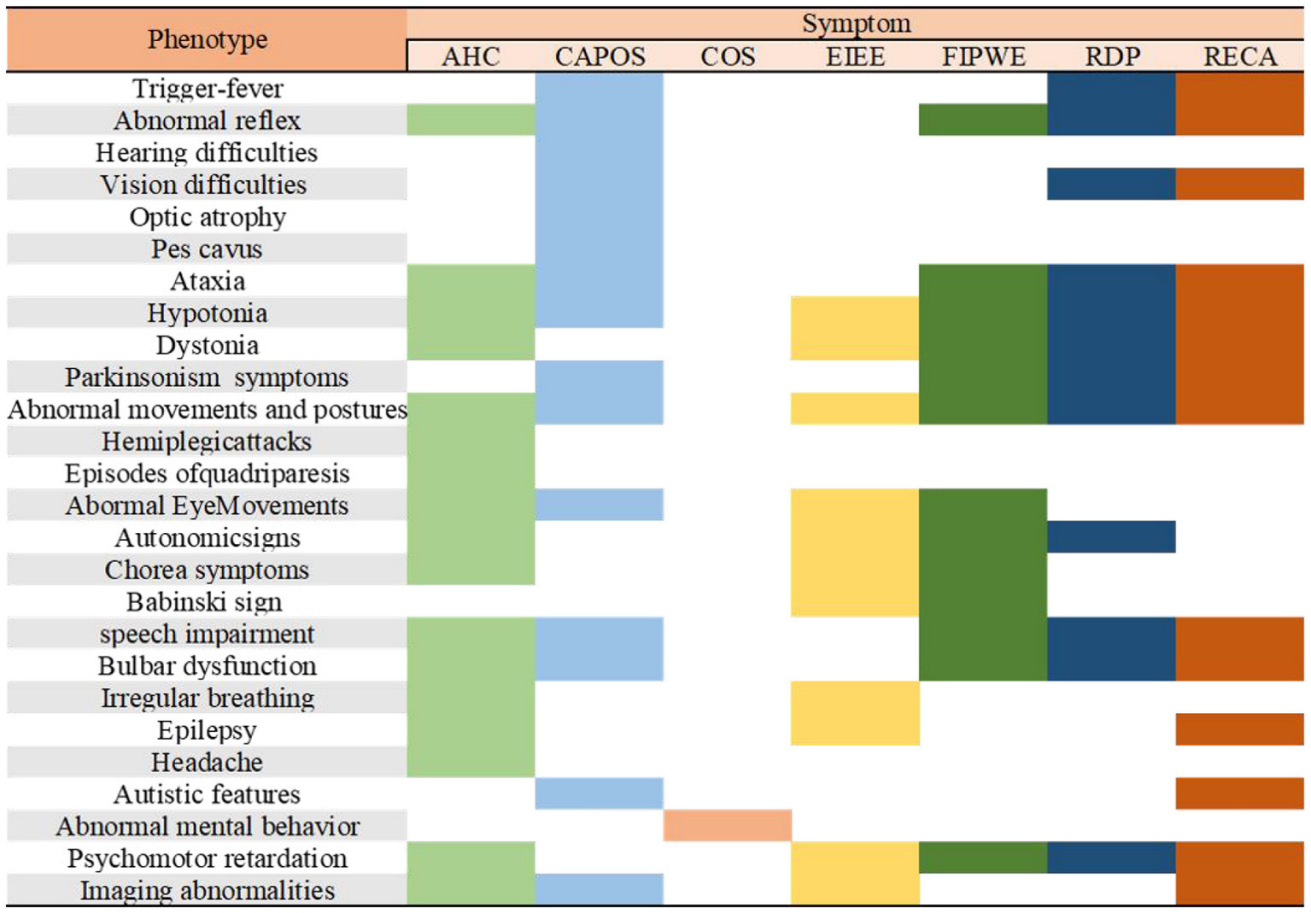
Characterization of the major phenotypes associated with *ATP1A3* mutations. AHC, alternating hemiplegia of childhood; CAPOS, cerebellar ataxia, areflexia, pes cavus, optic atrophy, and sensorineural hearing loss syndrome; epilepsy; COS, childhood-onset schizophrenia; EIEE, early infantile epileptic encephalopathy; FIPWE, fever-induced paroxysmal weakness and encephalopathy; RDP, rapid-onset dystonia-parkinsonism; RECA, relapsing encephalopathy with cerebellar ataxia.

The phenotypes of several other patients were ataxia-related, epilepsy, not relapsing episodes, nonprogressive encephalopathy with cerebellar ataxia, generalized hyperkinesia, movement disorders, early infantile epileptic encephalopathy (EIEE), childhood-onset schizophrenia (COS), auditory neuropathy spectrum disorder, and familial childhood-onset progressive cerebellar syndrome or with multiple overlapping phenotypes. The phenotypic characteristics and results of the clinical manifestations of other phenotypes are presented in Figure 1.

### Genetic characterization of the *ATP1A3* mutation

#### *ATP1A3* Mutation types and frequency of mutations in different clinical phenotypes

Five *ATP1A3* mutation types (complex rearrangements, missense, small deletions, small insertions, and splicing) were recorded in 902 cases. In this study, although no significant relationship was found between *ATP1A3* mutation types and phenotypes (*Fisher, p* = 0.219), some trends could be observed (Table 1). The vast majority of the clinical phenotypes were missense mutations (*n* = 868, 96.2%). Taken together, this analysis strongly predicted the pathogenic nature of missense mutations when these mutations were found in patients. Complex rearrangements were found only in AHC, and a small insertion was found in the RDP phenotype. The mutation with the highest frequency was p.D801N (*n* = 221, 24.5%), followed by p.E815K (*n* = 146, 16.2%), p.G947R (*n* = 59, 6.5%), and p.E818K (*n* = 57, 6.3%). A total of 87 mutations were found in AHC, and the highest mutation frequency was p.D801N (*n* = 216, 30.9%), followed by p.E815K (*n* = 144, 22.6%). The above two mutation frequencies accounted for more than half of the AHC phenotype, and p.G947R (*n* = 59, 9.2%) was also more common. None of the AHC-causing mutations were found in the clinical phenotype of the CAPOS syndrome, and most patients with AHC and RDP did not share the same mutations. In four patients with COS, an identical mutation was found in patients with AHC (p.E815K). In addition, 27 mutations were identified in patients with an RDP phenotype, of which p.I758S (*n* = 34, 28.6%) and p.T613M (*n* = 27, 22.7%) had higher mutation frequencies.

**Table 1:** Relationship between *ATP1A3* mutation types and phenotypes.

**phenotypes** *mutations Crosstabulation								
			**Complex**	**Missense**	**Small**	**Small**	**Splicing**	**Total**
			**rearrangements**	**Missense**	**deletions**	**insertions**		
phenotypes	AHC	Count	1	616	11	0	10	638
		% within phenotypes	0.20%	96.60%	1.70%	0.00%	1.60%	100.00%
		% within mutations	100.00%	71.00%	50.00%	0.00%	100.00%	70.70%
		% of Total	0.10%	68.30%	1.20%	0.00%	1.10%	70.70%
	CAPOS	Count	0	54	0	0	0	54
		% within phenotypes	0.00%	100.00%	0.00%	0.00%	0.00%	100.00%
		% within mutations	0.00%	6.20%	0.00%	0.00%	0.00%	6.00%
		% of Total	0.00%	6.00%	0.00%	0.00%	0.00%	6.00%
	COS	Count	0	4	0	0	0	4
		% within phenotypes	0.00%	100.00%	0.00%	0.00%	0.00%	100.00%
		% within mutations	0.00%	0.50%	0.00%	0.00%	0.00%	0.40%
		% of Total	0.00%	0.40%	0.00%	0.00%	0.00%	0.40%
	EIEE	Count	0	5	0	0	0	5
		% within phenotypes	0.00%	100.00%	0.00%	0.00%	0.00%	100.00%
		% within mutations	0.00%	0.60%	0.00%	0.00%	0.00%	0.60%
		% of Total	0.00%	0.60%	0.00%	0.00%	0.00%	0.60%
	FIPWE	Count	0	12	0	0	0	12
		% within phenotypes	0.00%	100.00%	0.00%	0.00%	0.00%	100.00%
		% within mutations	0.00%	1.40%	0.00%	0.00%	0.00%	1.30%
		% of Total	0.00%	1.30%	0.00%	0.00%	0.00%	1.30%
	Other types	Count	0	54	4	0	0	58
		% within phenotypes	0.00%	93.10%	6.90%	0.00%	0.00%	100.00%
		% within mutations	0.00%	6.20%	18.20%	0.00%	0.00%	6.40%
		% of Total	0.00%	6.00%	0.40%	0.00%	0.00%	6.40%
	RDP	Count	0	111	7	1	0	119
		% within phenotypes	0.00%	93.30%	5.90%	0.80%	0.00%	100.00%
		% within mutations	0.00%	12.80%	31.80%	100.00%	0.00%	13.20%
		% of Total	0.00%	12.30%	0.80%	0.10%	0.00%	13.20%
	RECA	Count	0	12	0	0	0	12
		% within phenotypes	0.00%	100.00%	0.00%	0.00%	0.00%	100.00%
		% within mutations	0.00%	1.40%	0.00%	0.00%	0.00%	1.30%
		% of Total	0.00%	1.30%	0.00%	0.00%	0.00%	1.30%
Total		Count	1	868	22	1	10	902
		% within phenotypes	0.10%	96.20%	2.40%	0.10%	1.10%	100.00%
		% within mutations	100.00%	100.00%	100.00%	100.00%	100.00%	100.00%
		% of Total	0.10%	96.20%	2.40%	0.10%	1.10%	100.00%

AHC, alternating hemiplegia of childhood; CAPOS, cerebellar ataxia, areflexia, pes cavus, optic atrophy, and sensorineural hearing loss syndrome; epilepsy; COS, childhood-onset schizophrenia; EIEE, early infantile epileptic encephalopathy; FIPWE, fever-induced paroxysmal weakness and encephalopathy; RDP, rapid-onset dystonia-parkinsonism; RECA, relapsing encephalopathy with cerebellar ataxia.

A total of four mutations were detected in CAPOS syndrome, and most mutations were p.E818K (*n* = 50, 92.6%), which was also detected in one RDP case. In addition, there were two cases of p.E831K: one was p.E819K, and the other was p.E820K in patients with CAPOS syndrome. p.R756H and p.R756C were detected in various clinical phenotypes, such as RDP, CAPOS syndrome, and AHC. Interestingly, a relationship was observed between the FIPWE phenotype and genotype, with the phenotypic mutation at position 756 arginine. Three related variation types were found, which included p.R756H, p.R756C, and p.R756L.

#### Distribution of *ATP1A3* mutations in the gene and protein structure

Analysis of missense mutations in 868 patients revealed that *ATP1A3* mutations were nearly distributed throughout the coding sequence, mainly in exons 17 (*n* = 376, 43.3%) and 18 (*n* = 242, 27.9%). Most of the mutation sites were located in the transmembrane region of the protein structure (*n* = 653, 75.2%), of which the sixth transmembrane region (TM6) of the *ATP1A3* protein was the most common mutation site (*n* = 479, 55.2%), followed by TM9 (*n* = 67, 7.7%), TM8 (*n* = 30, 3.5%), and TM5 (*n* = 27, 3.1%).

**Figure 2:**
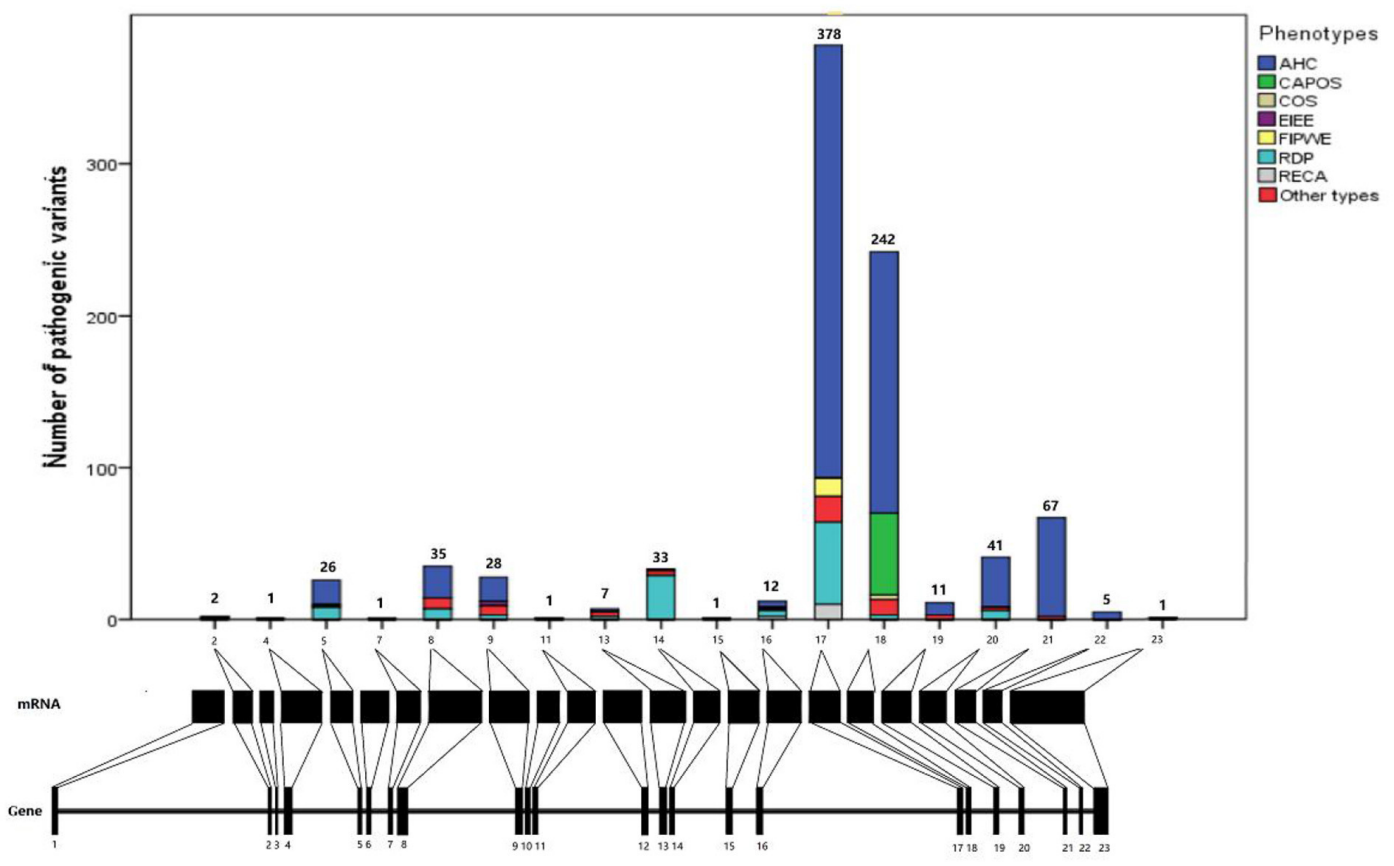
Distribution of *ATP1A3* mutations in gene and protein. AHC, alternating hemiplegia of childhood; CAPOS, cerebellar ataxia, areflexia, pes cavus, optic atrophy, and sensorineural hearing loss syndrome; epilepsy; COS, childhood-onset schizophrenia; EIEE, early infantile epileptic encephalopathy; FIPWE, fever-induced paroxysmal weakness and encephalopathy; RDP, rapid-onset dystonia-parkinsonism; RECA, relapsing encephalopathy with cerebellar ataxia.

As regards the affected functional domains, most *ATP1A3* pathogenic mutations affected ion-binding sites, ion pump transport efficiency, or enzyme phosphorylation. Some mutations also affected the cytoplasmic extension of transmembrane helix TM3–5, and a few pathogenic mutations affected the extracellular loop between TM7 and TM8. Essentially, no TM1 mutations were reported in the transmembrane helix region far from the ion-binding site (Figure 2).

### Therapeutic effect

In this retrospective analysis, flunarizine was used in 124 patients with AHC, of which 80 and 44 were effective and ineffective, respectively, with an effectiveness rate of ~64.5%. In addition, ATP administered orally can reduce the number of episodes in a patient, and some patients benefited from aripiprazole and topiramate. Trihexyphenidyl and amantadine were effective in a few patients with RDP. Some patients with the CAPOS phenotype exhibited partial improvement of symptoms following treatment with an antioxidant cocktail of steroids, amantadine, and acetazolamide. As relevant drugs were not recorded in many cases, the effects of the drug treatment should be further analyzed.

### Case report

#### Case 1

The patient was a 9-year-old girl with disease onset at the age of 3 months. She had two main forms of clinical presentation: (1) episodes of unilateral limb weakness, mostly on the right side and occasionally on the left side, which occurred easily after exercise or exertion and 5–7 days per month of cluster attacks, and (2) eye gazing to one side, absence of limb movement, and loss of consciousness, lasting from 10 min to several hours. Covering the eyes to make them fall asleep can shorten the duration of attacks. In addition, she exhibited developmental delay, motor dysfunction, lack of speech, and seizures. Treatment with levetiracetam, depakene, oxcarbazepine, clonazepam, and phenobarbital was ineffective.

**Figure 3:**
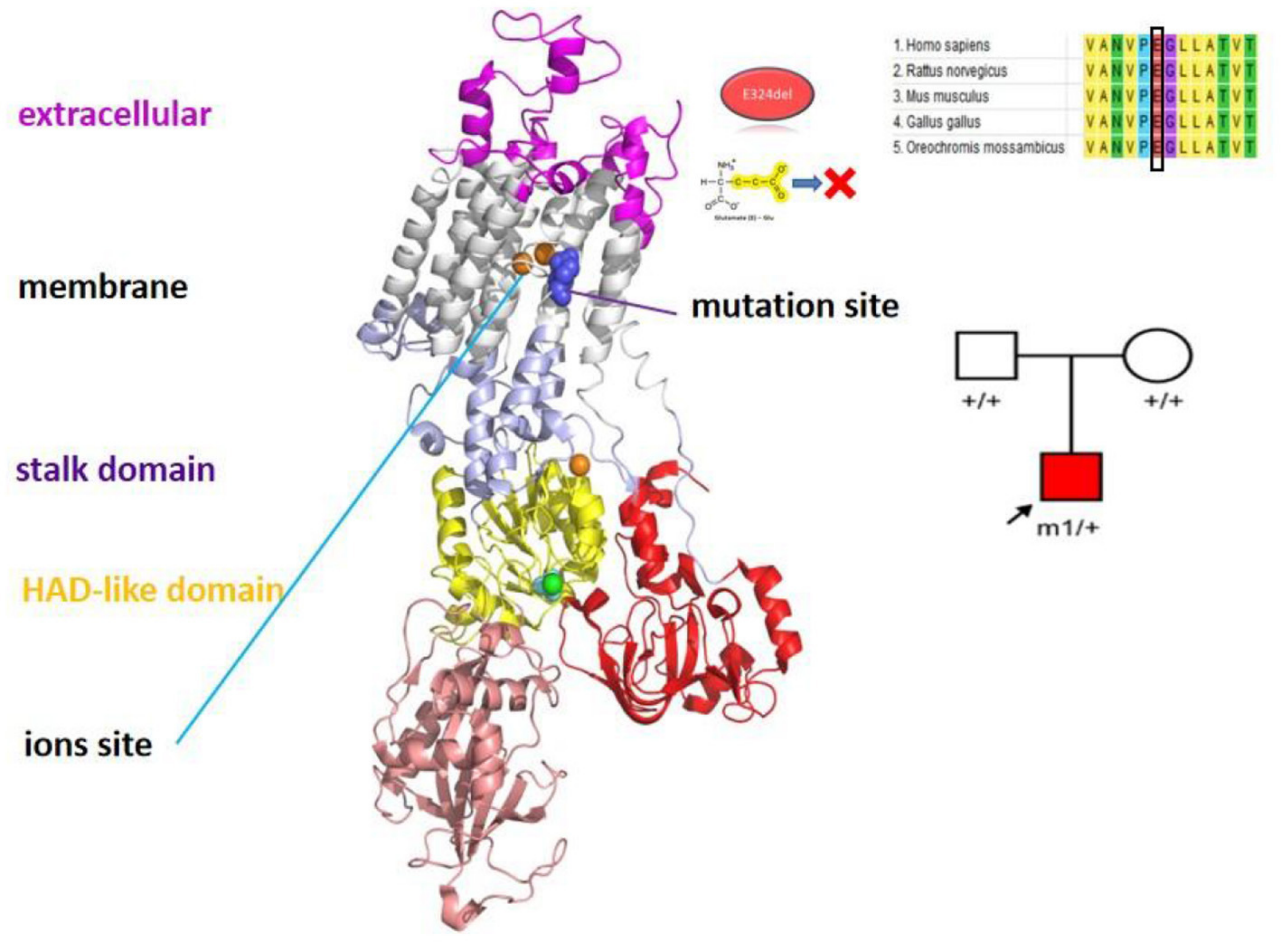
The position of the mutation sites *ATP1A3* p.E324del in the protein spatial structure.

Physical examination revealed poor comprehension and calculation, dysarthria, ataxia, and broad-based gait. Cranial MRI revealed atrophy of the cerebellar vermis. The result of the genetic tests revealed the heterozygous mutation c.971_973delAGG (p.E324del) in *ATP1A3* (Figure 3). After consultation at our hospital, she was diagnosed with AHC, and treatment with flunarizine was initiated, including adjustment of antiepileptic drugs to oxcarbazepine and depakene. At 9 months of follow-up, a significant reduction in attack episodes was noted.

#### Case 2

The other patient was a 118-day-old boy with disease onset at the age of 36 days. Four main forms of clinical presentation were observed: (1) He had episodes of rapid horizontal movement of the left eye during the waking state without limb tremors and confusion, which may resolve spontaneously. (2) He also presented with lateral eye gazing during the waking state, which lasted for 3–5 min, followed by rapid horizontal eye movements with or without limb weakness and decreased blood oxygenation, which resolved after a few seconds. (3) He also exhibited right upper limb straightening and trembling, right lower limb stiffness, left upper limb elbow flexion with trembling, and left lower limb flexion while awake. (4) He also exhibited cyanosis, mouth breathing, and blank stares while awake, followed by clonus-like shaking of the right limb and slight shaking of the head to the right. After a few minutes, his eyes were gazing to the left. He had nystagmus of the left eye, no activity of the right limb, clonus-like shaking of the left limb, and deafferentation tonic-like posture (upper limbs flexed and lower limbs straightened).

**Figure 4:**
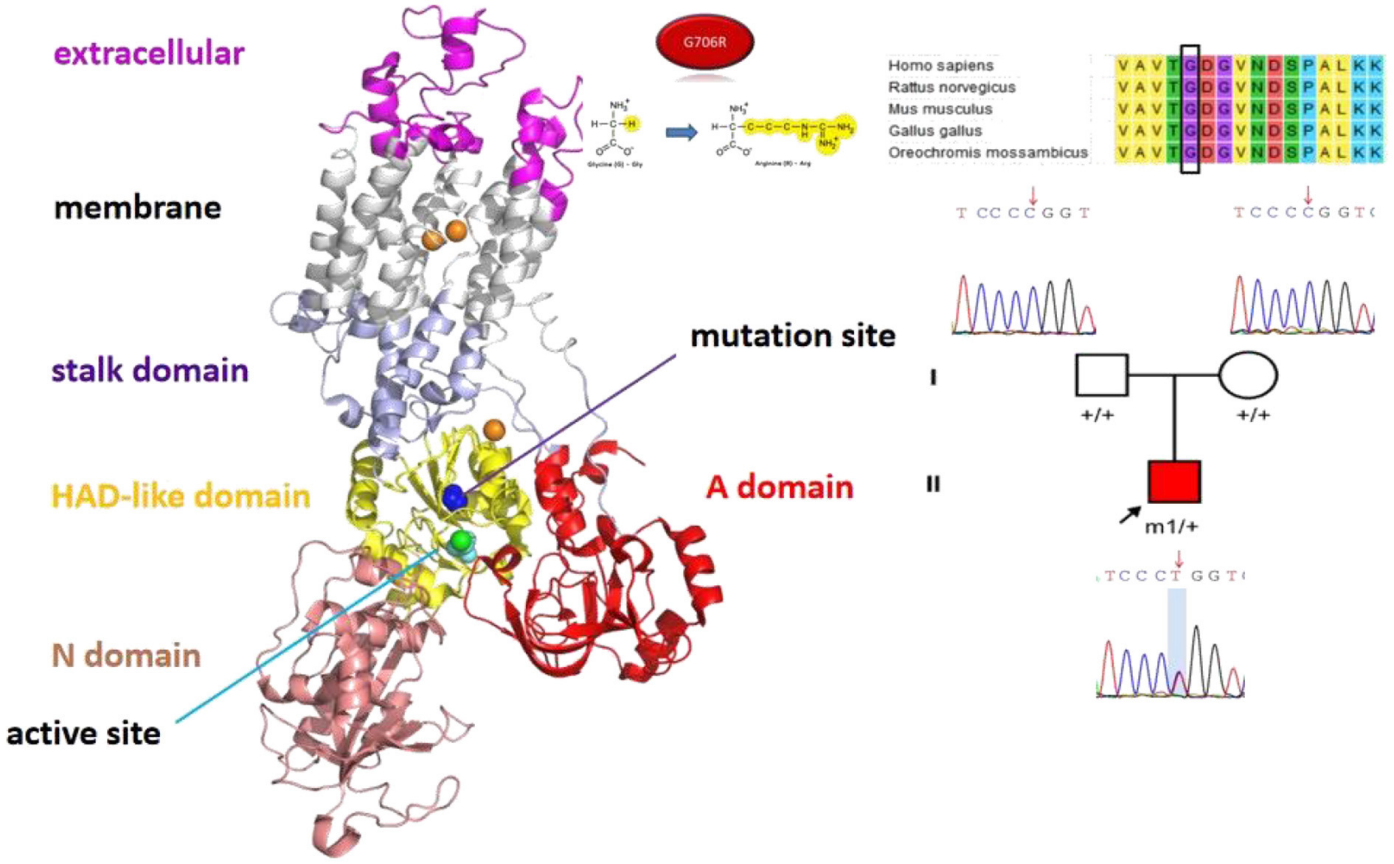
The position of the mutation sites *ATP1A3* p.G706R in the protein spatial structure.

He received levetiracetam, depakene, oxcarbazepine, clonazepam, and phenobarbital, but all were ineffective. Physical examination revealed that he could raise his head but unstably, and he could not keep track of light or sound. On cranial MRI, the bilateral cerebral white matter was not normally myelinated. The genetic test revealed heterozygous missense mutation c.G2116A (p.G706R) in *ATP1A3* (Figure 4). After consultation at our hospital, treatment with flunarizine was started, including dose adjustment of valproic acid. At 6 months of follow-up, the attack situation improved, and the duration and attack frequency became shorter.

## Discussion

### Clinical features and genotype–phenotype relationships of *ATP1A3* gene-related disorders

#### AHC

AHC was first reported by Verret and Steele in 1971 (5). Children with AHC not only presented with intermittent paroxysmal alternating hemiplegia and other characteristics but more variable neurological manifestations, including developmental delay, choreoathetosis, and dystonia. The onset age of the disease was mostly within 18 months, and the main symptoms were divided into episodic and interictal symptoms. The paroxysmal symptoms in children mainly included abnormal eye movements, alternating bilateral hemiplegic seizures, dystonia, and other types of seizures and others included autonomic symptoms and dyspnea. Most of the earliest symptoms of AHC were abnormal eye movements, which could be manifested as nystagmus, strabismus, gaze or eye movement, unilateral or bilateral involvement, and clear consciousness during the attack (6, 7). After the onset of hemiplegic symptoms, oculomotor abnormalities often appeared with hemiplegic episodes or sometimes alone. Recurrent alternating episodes of hemiparesis were the main symptom in children with AHC, with the peak age for the first hemiparesis being 6–7 months old. A hemiplegic attack could alternate between left and right, and one side of hemiplegia could have not been relieved and contralateral hemiplegia could occur, thus showing bilateral paralysis or quadriplegia or quadriplegia from the beginning of the attack. The duration of each hemiplegic attack varies from several minutes to several days, up to 14 days. Hemiplegia coud be relieved during sleep and could occur again 10–20 min after awakening. Dystonia often occured during the hemiplegia attack, but it can occur independently in a small number of children with AHC, often involving the limbs, neck, and trunk. It was mostly unilateral, with the child showing stiffness of one side of the body with extension of ipsilateral limbs, involving the neck, and often turning to one side of the head and neck to attack hemiplegia, with the eyeball turning to the same side. The incidence of epilepsy was 19–53%, which was prone to status epilepticus (6–8). There were many contributing factors to the paroxysmal symptoms, including mental stress, emotional agitation, environmental stimulation, contact with water (bathing, swimming, etc.), certain foods or smells, excessive or unusually strenuous exercise, and sleep factors (such as irregular sleep) (6, 7). Interictal symptoms included intellectual disability, motor retardation, and residual neurological dysfunction. Studies have reported that 92–100% of children with AHC had different degrees of developmental retardation, which manifested in cognitive function, large and fine motor skills, language expression, and mental behavior. Nervous system dysfunction mainly manifested as hypotonia, ataxia, choreographies, or hypotonia (6, 8).

Through this study, we identified the clinical manifestations of patients with AHC, including paroxysmal and interictal symptoms. The most common symptoms were abnormal eye movement, bilateral alternating hemiplegic attack, and dystonia. Some patients might have seizures, whereas others might have autonomic symptoms and dyspnea. In addition, these symptoms were often accompanied by other characteristic manifestations, such as psychomotor retardation, choreographies, and dystonia. Although most of the AHC cases were sporadic, a few AHC families have been reported, suggesting that genetic factors may be the cause of the disease (9). In 2012, two independent research teams confirmed that the *ATP1A3* gene was the main pathogenic gene of AHC. Previously, different research groups reported that the mutation rate of the *ATP1A3* in AHC cases was 78–100%, and the vast majority of mutations were *de novo* (10). The two more common gene mutations were p.D801N and p.E815K, and about 60% of the patients carried one of these two mutations (1). In addition, p.G947R gene mutations were also common. Compared with the phenotypes of these three common mutation types, the phenotype of p.E815K was the heaviest, followed by p.D801N and p.G947R. The patients with the p.E815K gene mutation exhibited earlier onset age, earlier onset of hemiplegia, more frequent attacks, more serious intellectual and motor impairment, and were more likely to have complications of epilepsy and status epilepticus (11). They also had a higher frequency of autonomic nerve dysfunction and were prone to respiratory dysfunction (12). In a study by Panagiotakaki et al., there was no significant difference in the frequency and duration of onset of hemiplegia, dystonia, and abnormal eye movement and the severity of mental and motor disorders between *ATP1A3* mutation-negative and -positive patients (12).

#### RDP

In 1993, Dobyns described the RDP pedigree with multi-member involvement for the first time. The main symptoms identified were dystonia with rapid onset, such as obvious dysphonia and dysphagia. RDP could also affect the upper limbs but the lower limbs were less affected. Parkinson's disease-like symptoms also occured, such as lack of expression, bradykinesia, and postural instability (13). The disease usually occured within a few hours but can persist from days to weeks. RDP was named after its clinical features, and the main clinical manifestations were (1) inducement; mostly occurring after physical or psychological stress, such as exercise, alcohol stimulation, mild head injury, fever, emotional tension, infection, or childbirth. (2) Prodromal symptoms; presented in some patients, usually with mild dystonia, involving the upper limbs but not the lower limbs. These were usually mild and limited to the distal part of the limbs. Systemic or trunk dystonia has not been reported. (3) The first attack; the age of onset usually ranges from 10 to 30 years old. There have also been cased with an earlier age of onset, with the youngest reported age of onset being 9 months, and onset at an earlier age was typically sudden within hours or days and in some cases within weeks (14). Medulla oblongata symptoms were more prominent. Typical manifestations were dysarthria, dysphonia, and dysphagia. Other main manifestations were involuntary movement and Parkinson's disease-like symptoms. Involuntary movement was characterized by systemic or partial dystonia, and Parkinson's disease-like symptoms were mainly bradykinesia and postural instability. Patients with these manifestations did not exhibit tremors, pill-rolling movements, or diurnal fluctuations, and did not respond to standard treatment for Parkinson's disease. The symptoms had obvious head–tail gradients, that was, the symptoms of the medulla oblongata were the most serious, followed by those of the upper limb then those of the lower limb. (4) The second attack; After acute onset, the symptoms tended to be stable and rarely showed a tendency to improve. Only a few patients showed symptoms of the lower limbs that could be slightly relieved, but the medulla oblongata symptoms and those of the upper limbs were not relieved. Some patients experience a sudden aggravation of symptoms 1–9 years after the first onset of the disease (15). (5) Non-motor symptoms; obvious social phobia, anxiety, depression, and schizophrenic tendencies have been observed in a pedigree from Ireland (16). In a retrospective study of patients with RDP, a higher prevalence of emotional disorders and mental diseases was observed (17). Another study showed that 29 patients with RDP were impaired in terms of attention, verbal fluency, coding task, visual memory, and speech learning tasks when compared with those in the control group, indicating that cognitive impairment was a part of the phenotype of RDP (18). In 1999, Kramer et al. determined that the 19q13 region was most likely to carry the RDP-related gene mutation (19). In 2004, some scholars reported that the pathogenic gene of the disease was the *ATP1A3* gene (20).

Although some scholars had suggested that there may be other pathogenic genes in RDP, *ATP1A3* remained the only known pathogenic gene (21). We counted 27 reported *ATP1A3* gene mutation types, among which I758S and T613M were the most common. No significant clinical phenotypic differences were found among different types of *ATP1A3* gene mutations. RDP usually occured in adolescence and adulthood, but it was also seen in childhood, even in infancy (11, 14). At present, however, no new gene mutations had been found in patients with childhood onset RDP.

#### CAPOS

In 1996, Nicolaides first reported three patients from the same pedigree. Their common clinical manifestations were early recurrent cerebellar ataxia, progressive optic nerve atrophy, and sensory hearing impairment, with varying degrees of areflexia and pes cavus, thus, the disease was named after the initials of these characteristic symptoms (22). Subsequently, Demos reported three families with the same characteristics and found that all of these patients had mutations in the *ATP1A3* gene (23). Heimer and Maas also reported the disease. In the reported cases of CAPOS syndrome, most of the symptoms were induced by febrile diseases (24, 25). The age of onset ranged from 6 months to 5 years old, with cerebellar ataxia, areflexia, optic nerve atrophy, and sensory hearing loss. Some patients also had pes cavus (23–25). Other symptoms reported in the attack period included manifestations of encephalopathy, such as myasthenia, disturbance of consciousness, abnormal eye movement, dysphagia, ophthalmoplegia, seizures, arrhythmias, and urinary tract symptoms (effective in the treatment of oxybutynin). Some of the symptoms of acute episodes, such as cerebellar ataxia, myasthenia, ophthalmoplegia, and disturbance of consciousness, can be relieved spontaneously within a few days to months, but reflex loss, hearing impairment, and optic nerve atrophy persist. Other reported sequelae of the nervous system included abnormal gait, laggard micromotor skills, fine motor dysfunction, nystagmus, and hypodystonia. With the increase of age, the sequelae can be progressively aggravated. Most of the patients were cognitively normal, and only one case was reported with cognitive impairment. One case was diagnosed with autism spectrum disorder. It should be noted that although the disease is named CAPOS syndrome, not all patients have pes cavus, and the prevalence rate of pes cavus in otherwise healthy individuals is about 10%. Therefore, some scholars have suggested that “P (pes cavus)” be removed from the name of the syndrome. In 2014, 10 cases of *ATP1A3* mutation in three families were reported by Demos et al., and they all carried the mutation of the *ATP1A3*, which was c.2452G > A. Glu818Lys. It was confirmed that the *ATP1A3* gene was the pathogenic gene of the disease (23). Subsequent studies on the genotypic–phenotypic correlation of CAPOS syndrome found that the vast majority of mutations were p.E818K (50 cases, 92.6%). The clinical phenotype caused by this mutation was that CAPOS rarely led to other clinical phenotypes, which was detected in one case of RDP, two cases of p. E831K, one case of p.E819K, and one case of p.E820K. Further studies are needed on how p.E.818K leads to the unique CAPOS syndrome rather than other *ATP1A3* gene spectrum diseases. There is only one mutation of c.2452G > A / p.Glu818Lys, and no other mutation sites have been reported. No asymptomatic carriers or CAPOS syndrome without *ATP1A3* gene mutation have been reported.

#### RECA

Dard et al. reported a 32-year-old female patient with a new phenotype caused by the *ATP1A3* gene mutation, which was characterized by recurrent encephalopathy accompanied by cerebellar ataxia, thus, it was named RECA (26). The patient had no previous history of epilepsy and no family history of neurological diseases. There were four episodes of acute encephalopathy in the course of the disease, which occurred at 22 months, 4, 6, and 32 years old, all of which were induced by febrile diseases. The symptoms were acute cerebellar ataxia, change of consciousness, hypodystonia, dysphagia, dysphasia, and pyramidal signs. The symptoms recovered completely after the first attack, but the cerebellar symptoms remained after the second attack, which was initially limb ataxia, followed by dysarthria and smooth eye movement abnormality. Systemic dystonia occurred after the fourth attack. Electromyography, electroencephalography, somatosensory-evoked potential, audiovisual evoked potential, ophthalmic examination, and metabolism-related examination showed normal results. Cranial MRI revealed mild atrophy of the cerebellar vermis. The results of Sanger sequencing using the patient's peripheral blood detected *ATP1A3* mutation c.2266C > T/p.Arg756Cys, which was a newborn mutation. Although CAPOS syndrome may also manifest as cerebellar ataxia and encephalopathy, it differs from CAPOS syndrome in loss of reflex, hearing impairment, and optic nerve atrophy. Moreover, the type of pathogenic mutation was different. At present, all the reported cases of CAPOS syndrome carried the mutation c.2452G > A/p.Glu818Lys, and in this patient, the gene mutation was Arg756Cys. Kanemasa et al. reported a patient with the Arg756Cys gene mutation, demonstrating a mixed phenotype of AHC, RDP, and CAPOS syndrome. Combined with this case, the mutation site Arg756Cys was speculated to have phenotypic heterogeneity (27). In 2017, some scholars described a group of patients with paroxysmal symptoms after a fever, including limb asthenia, ataxia, encephalopathy, dystonia, and involuntary movements. The phenotypic characteristics are similar to those of CAPOS syndrome, but no other manifestations, such as high foot arch, optic nerve atrophy, and sensorineural deafness, were noted. Therefore, it was considered a new disease type, named FIPWE. Analysis results presented a significant relationship between the phenotype and genotype, and the point variation led to the variation of arginine at position 756. At present, three types of related variants had been found, namely, c. 2267G > A (p.R756H), c. 2266C > T (p.R756C), and c. 2267G > T (p.R756L). Most of them are sporadic, but there were also familial cases of 28647130, and some cases may be chimerism. The genotype c. 2267G > A (p.R756H) was the most common. The decrease in muscle tone and strength was more apparent in patients with c. 2267G > A (R756H). Ataxia was more significant in patients with c. 2266C > T (R756C), whereas c. 2267G > T (p.R756L) may be between the two (14). Paciorkowski et al. reported two patients with early-onset epilepsy who carried the *ATP1A3* mutation and did not meet the diagnostic criteria of AHC (28). In the first case, the attack occurred 4 h after birth, with severe seizures and recurrent status epilepticus. Multiple antiepileptic drugs were ineffective, and the case was complicated with acquired microcephaly. Cranial MRI revealed progressive brain atrophy. The patient died of respiratory diseases at the age of 16 months. In the second case, the disease developed 6 weeks after birth. Epileptic seizures were often accompanied by prolonged apnea and were difficult to control. The patient had noticeable developmental retardation. In the second-generation sequencing, *ATP1A3* mutation was detected in both cases. The first case was c. 1073G > T/p.Gly358Val (G358V), and the second was c. 1088T > A/p.Ile363Asn (I363N). Both patients had severe seizures and obvious developmental lag during infancy, as well as early-onset epileptic encephalopathy and some AHC manifestations, such as abnormal eye movements, dystonia, and hemiplegia. Therefore, G358V and I363N mutations can lead to a mixed phenotype of early-onset epileptic encephalopathy and AHC. Seizures were found to occur in a Myshkin mouse model with I801N mutation in *ATP1A3*, indicating that the Na-K-ATP enzyme plays an important role in the control of epileptic electrical activity and seizures (29). Among patients with confirmed AHC, some develop seizures and status epilepticus in the neonatal period, and several studies have found that among the hot spot mutations of AHC, the E815K mutation can lead to epilepsy and severe developmental retardation during the neonatal period. Among the *ATP1A3* mutations, G358V, I363N, and E815K were related to early-onset epileptic encephalopathy. In addition, a study found that among the common mutations in the *ATP1A3*, rs8107107 is associated with hereditary generalized epilepsy, especially with generalized tonic–clonic seizures (30).

#### Treatment of AHC

Patients with AHC should receive drug therapy to prevent an attack during the non-acute episode phase, and flunarizine and topiramate can be used as preventive medications (16, 31). During an acute attack, symptoms can be controlled by the oral or intranasal administration of benzodiazepines. In addition, chloral hydrate, phenobarbital, and sleep inducers can relieve symptoms to varying degrees. A study reported that steroids can alleviate the clinical symptoms in the acute stage (32). During the disease course, first-line antiepileptic drugs can be considered for children with seizures. Antipsychotics may be considered in patients with residual psychiatric symptoms after the acute phase (16). At present, no study has examined other medical and surgical treatments, such as deep brain stimulation. Drugs, such as acetazolamide and melatonin, were found effective in patients with classic AHC (33).

#### Treatment of RDP

Currently, RDP has no specific treatment. In most reported cases, patients with RDP were sensitive to neither dopa drugs, such as levodopa and carbidopa, nor dopamine receptor agonists, such as praxol. Therefore, symptomatic treatment is the main treatment option for patients with RDP. Baclofen or high doses of benzodiazepines can slightly relieve symptoms of severe dystonia. Patients with a poor drug response can consider deep brain stimulation, but synchronous electrical stimulation of the bilateral globus pallidus is not recommended. In most patients, dystonia can be improved to varying degrees (34). In 2014, Fornarinao et al. reported a 13-year-old female patient with RDP who was sensitive to flunarizine therapy. In a recent case, flunarizine was reported to be effective in the treatment of RDP overlapping with the AHC phenotype (35). Patients with epileptic seizures, depression, anxiety, and other symptoms can be given routine symptomatic treatment. High doses of benzodiazepines may have partial efficacy in the treatment of RDP (16).

#### Treatment of CAPOS syndrome

CAPOS syndrome has no radical cure yet, and the long-term prognosis was closely related to the frequency of fever-related acute episodes. Acute episodes caused by infection or other febrile diseases should be avoided as much as possible. Acetazolamide and flunarizine hydrochloride are recommended to prevent an acute episode, but their efficacy is uncertain (23, 25, 36). Some patients have made remarkable progress in speech recognition after cochlear implantation (37).

Through the analysis of clinical correlations and distribution of mutations in this study, *ATP1A3* mutations were almost distributed in exons 17 and 18, and most of the mutation sites were located in the transmembrane region of the protein structure of which the TM6 of the *ATP1A3* protein was the most common mutation site, followed by TM9, TM8, and TM5. This suggests the importance of the structure and function of the transmembrane region of *ATP1A3*, which is closely related to the gene encoding the α3 subunit of Na^+^/K^+^-ATPase, a P-type cation transporter protein that plays a key role in maintaining the Na^+^ and K^+^ transmembrane electrochemical gradients in which the transmembrane region plays an important role. Their clinical features and disease course reflected the existing data on genotype–phenotype correlation, in which the transmembrane regions were associated with pathogenicity-associated genotypes and its therapy.

## Conclusions

At present, nervous system diseases related to *ATP1A3* mutations mainly are RDP, AHC, CAPOS syndrome, RECA. Despite the overlapping clinical phenotypes of each disease, they were not completely consistent, and there had been cases of *ATP1A3* mutations related to mixed or intermediate phenotypes among different diseases. Nervous system dysfunction due to *ATP1A3* mutations was characterized by a group of genotypic–phenotypic interrelated disease pedigrees with multiple clinical manifestations. Its diverse phenotypes involved paroxysmal symptoms of the nervous system, such as dystonia, hemiplegia, ataxia, epilepsy, abnormal eye movement, and bulbar symptoms, such as dysphagia and dysarthria, as well as permanent cognitive impairment, motor dysfunction, and emotional and behavioral disorders. The analysis of the *ATP1A3* genotype–phenotype relationship might help guide the diagnosis and management of nervous system diseases. With further investigation of *ATP1A3*-related diseases, the phenotypic spectra of the *ATP1A3* may be further expanded. Analyzing common characteristics of different diseases in the disease spectrum may provide clues for exploring the mechanism of *ATP1A3-*induced neurological dysfunction, providing the possibility of identifying new and effective treatments.

## Data availability statement

The raw data supporting the conclusions of this article will be made available by the authors, without undue reservation.

## Author contributions

LZ and SC conceived the idea to this paper. YL and ZS collected and analyzed the data and drafted the paper. CW, KZ, MY, and XL participated in the information registration and performed the statistical analysis. All authors read and approved the final manuscript.

## Funding

This work was funded by grants from the National Natural Science Foundation of China (82071447, 81571266, and 81771405) and the Sanming Project of Medicine in Shenzhen (No. SZSM201911003). The funders had no role in study design, data collection, and analysis, decision to publish, or preparation of the manuscript.

## Conflict of interest

The authors declare that the research was conducted in the absence of any commercial or financial relationships that could be construed as a potential conflict of interest.

## Publisher's note

All claims expressed in this article are solely those of the authors and do not necessarily represent those of their affiliated organizations, or those of the publisher, the editors and the reviewers. Any product that may be evaluated in this article, or claim that may be made by its manufacturer, is not guaranteed or endorsed by the publisher.
